# Non-Alcoholic Steatohepatitis and Hepatocellular Carcinoma: Implications for Lycopene Intervention

**DOI:** 10.3390/nu6010124

**Published:** 2013-12-27

**Authors:** Blanche C. Ip, Xiang-Dong Wang

**Affiliations:** 1 Nutrition and Cancer Biology Laboratory, Jean Mayer United States Department of Agriculture Human Nutrition Research Center on Aging at Tufts University, Boston, MA 02111, USA; E-Mail: blanche.ip@tufts.edu; 2 Biochemical and Molecular Nutrition Program, Friedman School of Nutrition Science and Policy, Tufts University, Boston, MA 02111, USA

**Keywords:** apolycopenoids, carotene oxygenases, inflammation, lipid metabolism, liver cancer, lycopene, NAFLD, NASH, oxidative stress, sirtuin

## Abstract

Increased prevalence of non-alcoholic fatty liver disease (NAFLD) is one of the consequences of the current obesity epidemic. NAFLD is a major form of chronic liver disease that is highly prevalent in obese and overweight adults and children. Nonalcoholic steatohepatitis (NASH) is the severe form of NAFLD, and uncontrolled inflammation as displayed in NASH has been identified as one of the key events in enhancing hepatic carcinogenesis. Lycopene is a non-provitamin A carotenoid and the pigment principally responsible for the characteristic deep-red color of ripe tomato and tomato products, as well as some fruits and vegetables. Lycopene’s innate antioxidant and anti-inflammatory properties have generated research interests on its capacity to protect against human diseases that are associated with oxidative stress and inflammation. In addition, differential mechanisms of lycopene metabolism including endogenous cleavage by carotenoid cleavage oxygenases (BCOs), generate lycopene metabolites that may also have significant impact on human disease development. However, it remains to be elucidated as to whether lycopene or its metabolites apolycopenoids have protective effects against obesity-related complications including inflammation and tumorigenesis. This article summarizes the *in vivo* experiments that elucidated molecular mechanisms associated with obesity-related hepatic inflammation and carcinogenesis. This review also provides an overview of lycopene metabolism, and the molecular pathways involved in the potential beneficial properties of lycopene and apolycopenoids. More research is clearly needed to fully unravel the importance of BCOs in tomato carotenoid metabolism and the consequence on human health and diseases.

## 1. Introduction

Liver cancer is the third leading cause of cancer-related deaths worldwide [1,2,3], and the most frequent and aggressive primary liver tumor is hepatocellular carcinoma (HCC) [1,2,3]. Prevalence of HCC is twice as high in males as in females, with very limited treatment options, and almost universally fatal within a year unless detected early [1,4]. Despite the gradual decline in all cancer incidences, HCC still tops the lists of cancers with increasing morbidity and mortality trends in the United States [1,4], with its prevalence doubled between 1983 and 2002 [5,6]. The risk for developing HCC is influenced by environmental factors, including viral induced hepatitis, alcohol consumption, and a recently defined factor, obesity [7]. HCC’s escalating morbidity and mortality trends parallel to the rising prevalence of non-alcoholic fatty liver disease (NAFLD), a chronic liver disease that is observed in 75%–100% of overweight and obese adults and children [8,9,10], and describes a range of related disorders that can progress in stages [11].

The earliest stage of NAFLD is hepatic steatosis, or lipids deposition in the cytoplasm of hepatocytes [10,11]. Hepatic steatosis can progress to the more aggressive form of NAFLD called nonalcoholic steatohepatitis (NASH) [8,10]. NASH is distinguished by the presence of hepatocyte injury (hepatocyte ballooning and cell death), infiltration of inflammatory cells, and may display collagen deposition [8,11]. NASH patients as compared to those with simple steatosis have a much greater risk for developing liver cirrhosis, a significant risk factor for HCC development [7]. Robust evidence suggests that NAFLD progression from steatosis to NASH and HCC involves a multi-step process, from liver damage initiation, followed by inflammation, cycles of necrosis, and regeneration [7]. Hepatic inflammation and injury in NASH are effective in activating hepatic stellate cells, which are specialized cells that involve in liver regeneration, but they can also promote cirrhosis by replacing hepatocytes with type I collagen-rich scar tissue [7,11]. This event results in an environment that is permissive to genetic modulations and exogenous impacts leading to neoplastic transformation [7,12]. HCC has extraordinary heterogeneity of genomic aberrations [13], but more than 90% of HCC develops based on chronic inflammation as displayed in NASH [13]. Such evidence highlights the critical role of hepatic inflammation in NAFLD associated hepatocarcinogenesis.

Approximately 30%–40% of HCC patients in the US have NAFLD [6]. These individuals incidentally have high prevalence for metabolic syndromes including obesity and type II diabetes mellitus [14], suggesting a strong association between metabolic syndrome, NAFLD and HCC development. The escalating obesity prevalence in both adults and children is a serious global public health concern [15], and accumulating epidemiological evidence support the positive association between obesity and HCC risk [16,17,18,19]. Calle *et al.* observed in a large prospective cohort study that individuals with a body mass index (BMI) greater than 35 has a relative risk for liver cancer mortality of 4.52 and 1.68 times greater than normal weight men and women respectively [18]. Two other population-based cohort studies from Sweden and Denmark yield similar conclusions [6]. Obesity is associated with a state of chronic low-grade inflammation that can induce hepatic inflammation, and can potentially play a role in NAFLD progression [7,12,17,18,20].

While targeting the root cause of metabolic syndrome including obesity may present the most effective prevention against NAFLD and HCC, it has been observed that caloric restriction on diet-induced obese mice was not effective in reversing the obesity-promoted tumorigenesis and associated signaling [21]. Therefore, potentially effective dietary preventions against this obesity-promoted tumorigenesis warrant investigation in easing public health burden. This review summarizes the recent data on the molecular mechanisms interconnecting metabolic syndrome, chronic inflammation, and HCC progression. This article also presents the accumulated evidence on how lycopene and its metabolites apolycopenoids may attenuate metabolic syndrome-associated hepatic injuries and HCC progression.

## 2. Molecular Mechanisms Associated with Metabolic Syndrome, Chronic Inflammation and HCC Progression

**Figure 1:**
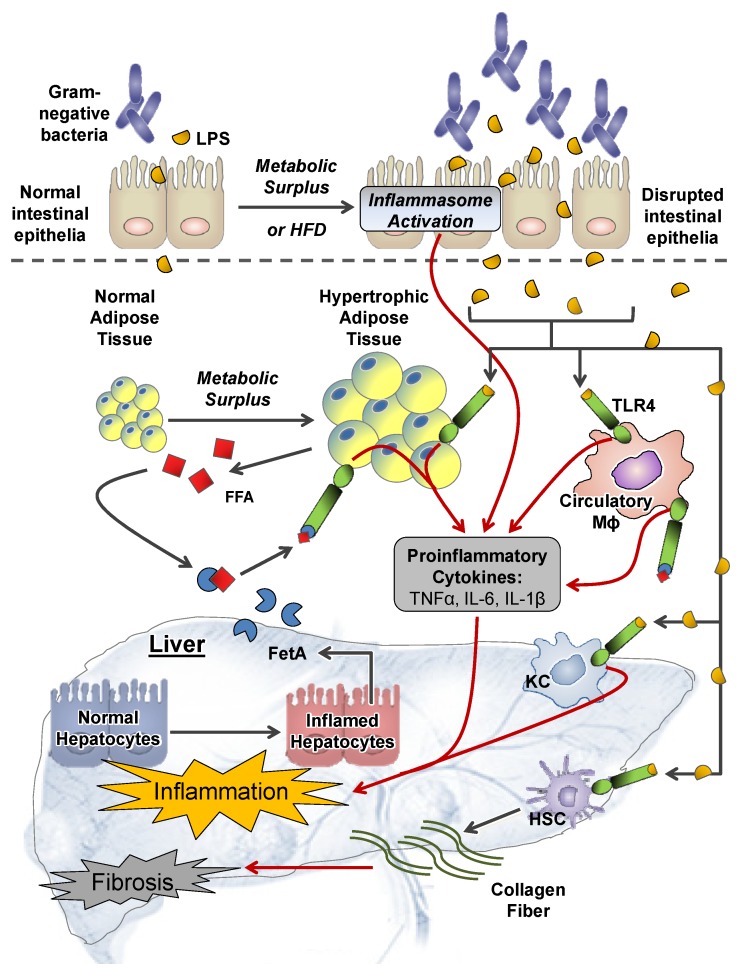
Mechanisms of extra-hepatic perturbations in non-alcoholic fatty liver disease (NAFLD) progression. Metabolic surplus and/or high fat diet (HFD) can disrupt the intestinal epithelia, leading to hepatic inflammation through promoting portal endotoxemia, increasing circulatory lipopolysaccharides (LPS) and activating the inflammasome. Circulatory LPS can stimulate proinflammatory cytokine secretion by macrophages (Mɸ) and adipocytes through toll-like receptor (TLR)-4-mediated signaling. LPS can also act on Kupffer cells (KCs) and hepatic stellate cells (HSCs) via TLR4 signaling to promote hepatic inflammation and fibrogenesis. Hepatic inflammation activates hepatocytes secrete fetuin A (FetA) into systemic circulation. Metabolic surplus stimulates adipocyte hypertrophy and the subsequent systemic release of free fatty acids (FFA). Circulatory FFA can associate with FetA released from the liver and activate TLR4-mediated proinflammatory signaling in adipocyte and Mɸ, creating a feed-forward mechanism in promoting further systemic and hepatic inflammation. IL: interleukin.

**Figure 2:**
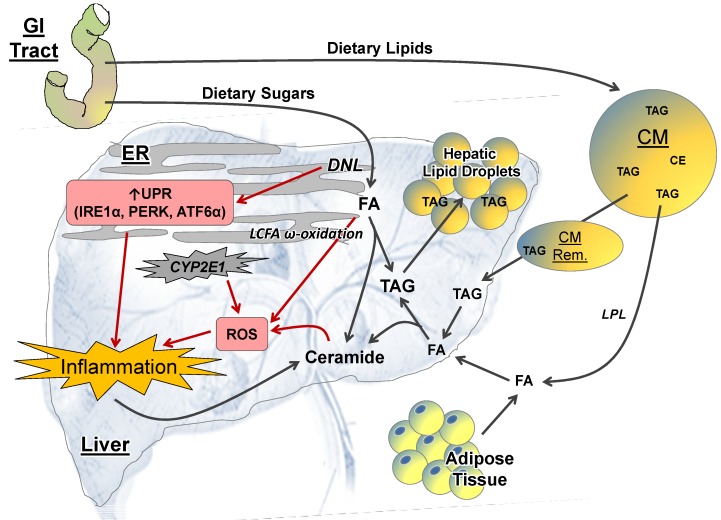
Mechanisms of intra-hepatic perturbations in non-alcoholic fatty liver disease (NAFLD) progression. Increase in dietary lipids elevates triglyceride (TAG) and cholesterol delivery to the liver by chylomicrons (CM) and CM remnants (Rem.). Excess TAG is stored in the liver and can promote hepatic steatosis. TAG repackaging involves generation of fatty acids (FAs) and ceramide. Accumulation of ceramide can disrupt mitochondrial function and induce reactive oxygen species (ROS) production, which subsequently leads to hepatic inflammation. Excess hepatic long-chain FA (LCFA) can undergo ω-oxidation in the endoplasmic reticulum (ER), and result in generation of ROS. ROS production can also be stimulated by cytochrome (CYP) P450 enzyme activation. Elevated dietary sugars can stimulate *de novo* lipogenesis (DNL) to generate FA, and stimulate ER stress-mediated unfolded protein response (UPR), leading to hepatic inflammation. ATF6α, activating transcription factor 6α; CE, cholesterol esters; GI, gastrointestinal; IRE1α, inositol requiring 1α; LPL, lipoprotein lipase; PERK, PKR-like ER kinase.

NAFLD and NASH associated hepatic inflammation involves mechanisms that stemmed from both extrahepatic and intrahepatic perturbations. To find potential molecular targets for disease prevention and treatments, it is essential to dissect the molecular mechanisms by which obesity promotes liver inflammation and injuries, and to understand how these mechanisms integrate to promote NASH and HCC development. Schematics for extrahepatic and intrahepatic perturbations are displayed in Figure 1 and Figure 2 respectively.

### 2.1. Extrahepatic Perturbations

#### 2.1.1. GI Tract

Consumption of high fat diets (HFD) can promote hepatic inflammation by disrupting the intestinal barrier, thereby allowing increased translocation of bacteria and related antigens into the systemic circulation (Figure 1) [22,23,24,25,26,27]. Liver receives a unique blood supply via the portal system connecting itself to the GI tract, exposing liver cells to nutrients as well as bacterial components that are translocated [28]. Increased intestinal permeability is common among patients with chronic and advance liver disease [29,30,31], and can be associated with alterations and/or increased in gut microflora population [22,26]. Intestinal disruption can elevate portal endotoxemia by up to three-fold in healthy individuals on HFD [32], and 6 to 20-fold in individuals with NAFLD [33]. Portal endotoxemia can sensitize hepatic stellate cells and resident macrophages called Kupffer cells (KCs) to bacterial endotoxins including lipopolysaccharides (LPS), leading to liver fibrosis and cirrhosis (Figure 1) [34,35,36,37,38,39,40,41]. The potential mechanisms involved include endotoxin-mediated activation of toll-like receptor (TLR) 4 pro-inflammatory signaling [35,42], and the innate immune signaling complex called the inflammasomes (Figure 1) [43].

TLR4-mediated signaling can activate the hepatic pro-inflammatory nuclear factor-κB (NF-κB) signaling cascade to induced hepatic inflammation and oncogenic interleukin 6 (IL-6)-signal transducer and activator of transcription 3 (STAT3) pathway [44,45]. Detail mechanistic activation of NF-κB signaling and subsequent pro-inflammatory response in liver diseases has recently been reviewed [7,46]. Inflammasomes are a group of intracellular multimeric protein complexes that mediate autoactivation of caspase 1 when activated by endotoxin and other particles that have surface expression of foreign epitopes [43]. Activation of caspase 1 by cleavage results in the maturation and secretion of the pro-inflammatory cytokines IL-1β and IL-18, leading to hepatic inflammation. Caspase 1 activation can also inactivate sirtuin 1 (SIRT1) by cleavage [47], where SIRT1 is a NAD^+^-dependent protein deacetylase with anti-inflammatory properties [48,49,50,51,52], and it is recognized to protect against obesity-induced hepatic steatosis and inflammation [48,53,54,55,56]. IL-1β expression has recently been shown in mice to promote senescence-associated secretory phenotype (SASP) in hepatic stellate cells that facilitates chemical carcinogen-induced HCC development [57].

#### 2.1.2. Adipose Tissue

Obesity disrupts the dynamic role of adipocyte in energy homeostasis, resulting in extrahepatic perturbations including systemic inflammation and altering adipokine signaling (Figure 1) [20]. Under the obese state, adipose tissue expansion signals immune cells to infiltrate into the tissue and secrete pro-inflammatory cytokines tumor necrosis factor alpha (TNFα) and IL-6 [20]. These pro-inflammatory cytokines from adipose tissue can be released into systemic circulation along with free fatty acids (FFA) from metabolic excess [12,17,58,59,60,61], perturbing the function of other peripheral tissue including the liver [58,62].

Systemic elevation in FFA and cytokines can act upon hepatocytes through interacting with their respective receptors including the TLR4 [44,45]. Hepatic NF-κB signaling activation can trigger the systemic release of hepatic fetuin A (FetA) [44,45,63], a liver secretory glycoprotein elevated in NAFLD [63,64]. FetA associated with FFA can stimulate inflammatory cytokine production from adipocytes and macrophages via TLR4 receptors [44,45,63], creating a feed-forward mechanism to promote further systemic inflammation (Figure 1). Elevated systemic FFA especially those that are saturated can also activate the Jun kinases (JNKs) pathway especially in myeloid cells to induce pro-inflammatory cytokines production [58,65]. JNKs are members of the mitogen-activated protein kinase (MAPK) signaling group, and are activated by physical stress and receptor mediated mechanisms including the TNF receptor 1, TLR2 and TLR4 [66,67]. Detail mechanistic activation of MAPK signaling and subsequent pro-inflammatory response in liver diseases has been extensively reviewed [7]. Recently, saturated FA was found to activate JNK signaling by decrease membrane fluidity, leading to subsequent clustering and activation of c-Src that activates the JNK pathway [68]. This evidence suggests that alterations in dietary FA composition may modulate hepatic pro-inflammatory signaling.

Circulatory TNFα and IL-6 are believed to be the pivotal cytokines involved in obesity-associated hepatic inflammation [20,69,70,71]. Systemic TNFα can prime hepatic KCs and hepatocytes receptor-mediated mechanisms [72,73], resulting in the activation of pro-inflammatory signaling including the Activation Protein-1 (AP-1), NF-κB and JNK [72,73]. JNK activation can induce many physiological and pathophysiological processes including apoptosis, cell proliferation, cell migration and cytokine production. JNK activation was also associated with Ras-MAPK signaling activation that leads to the phosphorylation of retinoid X receptor (RXR)-α [74], a key step in HCC development [75,76,77].

#### 2.1.3. Other Systemic Perturbations

Elevated expressions of circulatory growth hormone and factors including insulin and IGFs have been associated with metabolic syndrome [78,79]. The signal transduction networks of insulin and IGFs both play critical roles in promoting neoplasia in various tissues, whereas circulatory IGF-binding proteins (IGFBPs) attenuate the bioactivities of IGFs [78,79]. Insulin and IGFs can mediate mitogenic signaling through receptor-mediated mechanisms, thereby activating Ras-MAPK and the phosphatidylinositol 3-kinase (PI3K) and Akt pathways [78,79]. These pathways are important regulators of mammalian cell proliferation and survival, and are often dysregulated in human cancers [78,79]. It remains controversial as to whether obesity is associated with increased IGFs levels. Some epidemiological studies have observed higher systemic IGF1 and IGF2 in obese individuals with T2DM [67,68]. However, obesity also reduces the release of growth hormone that controls hepatic IGF synthesis [69]. The association between IGF and BMI from the European Prospective Investigation into Cancer and Nutrition (EPIC) study was non-linear, where those with a BMI of 26–27 had the highest circulatory IGF1 concentration [80]. However, a linear negative association between IGFBP1 and BMI was observed [80], suggesting that dietary components that induce IGFBP expression may still be effective in reverting oncogenic effects of IGFs.

Other hallmarks associated with NAFLD are systemic lipid markers that are also risk factors of atherosclerosis, including elevated triglyceride (TG), cholesterol (CHOL), low density lipoprotein (LDL) and oxidative stress [81]. The elevated LDL in the presences of oxidative stress can result in the increased presences of oxidized LDL (oxLDL) [81]. oxLDL is an emerging factor that is associated with hepatic inflammation, and may promote the progression from steatosis to NASH [82,83]. Under physiological conditions, myeloid cells including macrophages can internalize excess lipoprotein-derived cholesterol by receptor-mediated systems [82,83]. When LDL becomes highly oxidized, these particles may display structures that are similar to pathogen-associated epitopes that increases their uptake by macrophages, partially through scavenger receptors A (SR-A) and CD36 [84]. Internalization of these oxLDL by macrophages has been postulated to accumulate in the lysosomal compartment due to poor degradation, leading to presence of cholesterol crystals [81,85,86]. The presence of these internal cholesterol crystals, along with oxidation-specific epitopes of LDL can be seen as damaged associated molecular patterns (DAMPs) and can activate the inflammasomes [87]. Apart from stimulating the inflammasome, accumulation of oxLDL-derived cholesterol can also induce the activation of NF-κB signaling [88,89]. These described oxLDL-stimulated pro-inflammatory effects are well-documented on circulatory macrophages, but emerging evidence have provided support that similar events do occur in KCs [85]. Isolated KCs from mice with early NASH has been shown to predominately accumulate cholesterol and cholesterol crystals specifically in the lysosomes, and displayed pro-inflammatory phenotypes [85,86,90]. Hepatic inflammation observed in these mice was associated with the increased cholesterol storage inside lysosomes of KCs [86,91]. Intravenous injection of oxLDL for 2 weeks induced severe hepatic inflammation as displayed in NASH in mice fed HFD for 23 weeks, as compared to PBS injected HFD-fed mice [92]. *SR* and/or *Cd36* deletion reduced hepatic inflammation and the development of NASH [86,91]. It remains to be elucidated as to whether oxLDL-induced hepatic inflammation stems mainly from affected circulatory macrophages or KCs.

### 2.2. Intrahepatic Perturbations

#### 2.2.1. Lipid Metabolism

Hepatic lipid accumulation in NAFLD results from disturbance in lipid homeostasis, and can contribute to NASH progression through intrahepatic perturbations by multiple mechanisms (Figure 2) [93,94]. Elevated FFA as described previously can up-regulate the expression of hepatic FA transporter, leading to hepatic lipid accumulation [93,94]. Metabolic excess from diets especially rich in carbohydrates can promote hepatic lipid accumulation through activating hepatic lipogenesis including *de novo* synthesis of FAs [93,94]. FA synthesis is catalyzed by acetyl-CoA carboxylase (ACC) and fatty acid synthase (FAS), enzymes that are regulated by nuclear receptors including PPARs, FXR and SREBP-1c/CHREBP [95,96,97,98]. Donnelly *et al.* observed that NAFLD patients have dysregulated *de novo* lipogenesis (DNL), where DNL is significantly elevated at fasting state, contributing ~25% of lipids in very low density lipoproteins as compared to ~5% in healthy individuals [99]. Analysis of human normal liver, HCCs and corresponding surrounding non-tumor liver tissue showed Akt-signaling activation in non-neoplastic surrounding livers and HCC when compared with normal livers [100]. This observation is associated with increased hepatic CHOL, TG, and the progressive induction of lipogenic proteins including ACC, FAS and SCD1 [100], suggesting the potential tumorigenic effects of lipogenesis and the association with Akt activation.

Increased hepatic ceramide has also been associated with NAFLD progression [101,102,103], where liver is the key site for ceramide synthesis [102]. Ceramides are members of the sphingolipid family that are integral to the membrane bilayer structure, but also have cell-signaling properties [102]. Three different pathways are responsible for ceramide synthesis: *de novo* synthesis, a sphingomyelinase pathway, or a salvage pathway [102]. A diet high in fat, specifically rich in saturated fatty acids, can up-regulate the rate-limiting enzyme involved in ceramide *de novo* synthesis (Figure 2) [104,105]. Other perturbations exhibited in NAFLD including elevations in circulating LPS and FFA, as well as increased oxidative stress and inflammation can also promote ceramide synthesis [101,106,107,108].

Numerous pathways have been elucidated for ceramide’s role in NAFLD progression. Specifically with hepatic inflammation, ceramide can lead to mitochondrial dysfunction [102], resulting in the subsequent elevation in ROS (Figure 2). Increased mitochondrial generation of ROS can promote hepatocyte apoptosis, and a further recruitment of inflammatory cells into the liver, creating a vicious cycle to sustain and amplify hepatic inflammation [102,109,110,111].

#### 2.2.2. ER Stress

Elevated synthesis of lipogenic enzymes demands excess work of the endoplasmic reticulum (ER) for proper protein folding [112,113,114], hereby inducing ER-stress. Hepatic DNL has been impeded in promoting hepatic inflammation through activation of ER stress-mediated unfolded protein response (UPR; Figure 2) [112,113,114]. UPR consists of three distinct pathways regulated by ER membrane-bound proteins: inositol requiring (IRE) 1α-X-box binding protein 1 (XBP1) system, PKR-like ER kinase (PERK)-eukaryotic initiation factor (eIF) 2α signaling, and activating transcription factor (ATF) 6α [112,113,114]. Increased activation of IRE1α and ATF6α signaling in HCC tissue were correlated with increased severity of HCC histological grading [115], and were associated with greater expression of the carcinogenic glucose regulated protein 78/binding immunoglobulin protein (GRP78/BiP) that is overexpressed in human HCC [115]. Activation of the PERK and IRE1 pathways can promote NF-κB mediated pro-inflammatory response, whereas PERK-signaling in tumors can promote cellular growth through activation of Akt [116,117,118]. ER stress can also exacerbate lipid accumulation by activating lipogenesis through SREBP-1c [119], creating a vicious cycle to sustain the pro-inflammatory state.

#### 2.2.3. ROS and CYP-P450 Enzymes

FA oxidation occurs within mitochondria, peroxisomes and the ER [93,94]. β-Oxidation that occurs in the mitochondria is effective in generating cellular energy, and maintaining lipid homeostasis [93,94]. Unlike short- or median-chain FAs, long-chain FAs (LCFA) in the cytosol require carnitine palmitoyltransferase-1 (CPT-1) for their translocation into the mitochondria [93,94]. Malonyl-CoA, an early intermediate of DNL synthesized by ACC, is an allosteric inhibitor of CPT-1 [120], thus elevated DNL can inhibit FA β-oxidation and promote excess hepatic lipids in NAFLD [121]. Hepatic lipid overload can also promote LCFA ω-oxidation that occurs in the ER and induce the generation of ROS (Figure 2) [122], which is a key factor for NASH progression from steatosis [123].

ROS are toxic to macromolecules (lipids, proteins, RNA and DNA), and may facilitate cancer initiation [123]. Dysregulation in the balance between ROS generation and removal will lead to oxidative stress, which promotes inflammation, apoptosis and necrosis [123]. ROS can also be generated by the activation of CYP-P450 enzymes (Figure 2) [123]. The increased CYP-P450 protein expression and activity have been observed in obesity and NAFLD in both humans and rodents [124]. The CYP-P450 superfamily consists of a group of enzymes with multiple functions. The main role of CYP-P450 protein in the liver includes the metabolism of xenobiotics (drugs, toxins, carcinogens) and endogenous substrates (FAs and steroids) [123,124]. CYP2E1 is a CYP-P450 enzyme that is mainly expressed in the liver, highest in hepatocytes, and has been identified as one of the relevant risk factors in NAFLD pathology [123,124,125]. CYP2E1 overexpression in a transgenic mouse model induced hepatic steatosis and injury [126], whereas application of CYP2E1 antibody inhibited hepatic lipid peroxidation in murine NASH model [127]. Further, *Cyp2e1* knockout mice are protected from HFD-induced insulin resistance [128,129], providing evidence to suggest that CYP2E1 does play a role in NAFLD progression and metabolic syndrome.

#### 2.2.4. Inflammation and TLR4

Innate immunity involving TLRs provides the first line of defense against microbial invasion. The anatomical connection between the intestine and liver renders hepatic cells being constantly exposed to intestinally-derived microbial components [42]. Liver cells specifically hepatocytes play a major role in the uptake of these intestinal-derived microbial components including Gram-negative bacterial LPS, and their subsequent clearance from systemic circulation via bile secretion [130]. TLRs are pattern-recognition receptors that recognize microbial components, including pathogen-associated molecular patterns (PAMPs) such as LPS [131]. Activation of TLRs subsequently elicits innate immune response including pro-inflammatory signaling (Figure 1). Under normal physiological conditions, only small amounts of intestinally-derived microbial components are translocated to the liver, and this low baseline activation of TLRs do not induce liver inflammation or injury [132]. However, disruption of the intestinal barrier from metabolic surplus and HFD can increase hepatic exposure to PAMPs [22,32,33], leading to an exacerbated activation of TLR-mediated signaling.

TLR4 mediates innate immune signaling by recognizing the lipid immunogenic proportion of LPS, but TLR4 activation by LPS requires co-receptors CD14 and MD-2, as well as the facilitation of LPS-binding protein (LBP) that acts as a soluble shuttling protein [130]. Different TLRs elicit specific biological response due to their differential involvement of Toll/interleukin 1 receptor (TIR) domain-containing adaptor molecules, including the MyD88 and TIR-domain-containing adapter-inducing interferon-β (TRIF) [131]. MyD88-dependent signaling involves both MAPK and NF-κB signaling activation, leading to the production of pro-inflammatory cytokines TNFα and IL-6 [130,131]. TRIF-activation can induce an alternative pathway, leading to the activation of the transcription factors IRF3 and NF-κB and the subsequent production of Type I interferon (IFN) and inflammatory cytokines [130,131].

TLR4 is expressed in all types of liver cells [130]. KCs are among the first liver cells to be exposed to intestinal-derived microbial components, due to their localization in the liver sinusoid [130]. KCs mediate majority of cytokine and chemokine production after LPS stimulation (Figure 1) [35], and results in the induction of oxidative stress, as well as fibrogenic responses including expression of transforming growth factor beta 1 (TGFβ1), matrix metalloproteinases (MMPs) and platelet-derived growth factor [36]. IL-12 and IL-18 released by KCs can activate hepatic natural killer cells, leading to the synthesis and the release of IFN-γ [133]. However, LPS-stimulated KCs also secrete anti-inflammatory cytokine IL-10 and can contribute to the downregulation of pro-inflammatory response [134]. Activation of TLR4 in hepatic stellate cells stimulates release of pro-inflammatory cytokines and extracellular matrix (ECM) production [36], which are critical steps in promoting hepatic fibrogenesis (Figure 1).

## 3. Effects of Tomato and Lycopene Consumption against NASH and HCC

### 3.1. Tomato Effects

Observational and clinical studies have shown that dietary intake of tomatoes is associated with reduced risk for human cancers at numerous sites as reviewed previously [135,136,137], and this association remains to be explored with liver cancer. However, results yielded from epidemiological studies are often difficult to conclude whether the observed chemopreventative effects are tomato or lycopene-dependent. This issue originates from the existing methodology in estimating dietary intake of tomatoes and/or lycopene, of which includes food frequency questionnaire and serum analysis [135,137]. Clinical trials have been conducted to examine the effects of tomato products and lycopene containing supplements on various cancers [135,136]. Similar to epidemiological studies, the clinical studies that utilized tomato products, as well as lycopene-containing supplements that often contain other phytonutrients (phytoene, phytofluene, β-carotene, and tocopherols), are difficult to isolate lycopene’s beneficial effects. Therefore, human interventions and animal studies that compare the effects between isolated lycopene and the whole food tomato are essential in differentiating their potential beneficial effects against liver diseases. Tomatoes by definition provide a high source of vitamin A and C, but they also consist of phytochemical including carotenoids and flavonoids. Lycopene is the most abundant carotenoid found in tomato, tomato products and other red fruits, and these food products have also been shown to contain lycopene metabolites [138]. With respect to liver diseases, we have previously shown that tomato extract supplementation was more protective against HFD-induced hepatic inflammation than lycopene [139]. Clearly, further investigations are needed to examine whether nutrients in tomatoes may work additively or synergistically in protecting against liver diseases.

### 3.2. Lycopene Effects

Studies suggest that lycopene can be one of the important bioactive compounds responsible for reducing chronic diseases risk including cardiovascular disease and cancer, which have been extensively reviewed [136,140,141]. The evidence that supports the anti-carcinogenic properties of lycopene stemmed from various epidemiological studies, where high intake of lycopene-rich fruits and vegetables is associated with cancer mortality from all sites [142,143]. A significant inverse relationship was also found between serum lycopene and the risk of a number of cancers including those of gastrointestinal tract origin [137,144,145]. This relationship is yet to be discovered between lycopene and liver cancer due to the low disease prevalence [137,144,145,146], except for the risk of alcohol-induced HCC [147]. But importantly, adverse effects have not been associated with consuming high levels of tomatoes or lycopene [137,144,145]. In regards to liver cancer risks, NASH patients have been shown to have significantly reduced plasma lycopene [148], suggesting the potential interactions between low lycopene status and the development of liver diseases [148].

*In vitro* and *in vivo* experiments have demonstrated that lycopene may have chemopreventative effects against liver cancer development. Lycopene treatments on hepatoma cell lines showed inhibitory effects on cellular growth, migration and invasion [140,149,150]. Dietary lycopene inhibits liver-specific carcinogen diethylnitrosamine (DEN)-induced preneoplastic lesions in the rat liver [139,151,152,153], and effective in reducing NASH-promoted, DEN-initiated hepatocarcinogenesis in rats [139,151,152]. Lycopene supplementation in rats that develops spontaneous liver tumors also significantly reduced the GST-P-positive areas in HCC lesions, a marker for tumor promotion [153]. However, the primary outcomes for these rat studies were hepatic preneoplastic lesions that can potentially develop into liver tumors. There are currently no published *in vivo* studies to demonstrate whether lycopene can effectively reduce liver tumor development and progression. The effect of lycopene against HCC may also vary depending on the HCC experimental models, where dietary lycopene has failed to protect against liver cancer in rats that develops this cancer spontaneously [154].

### 3.3. Lycopene Metabolism

**Figure 3:**
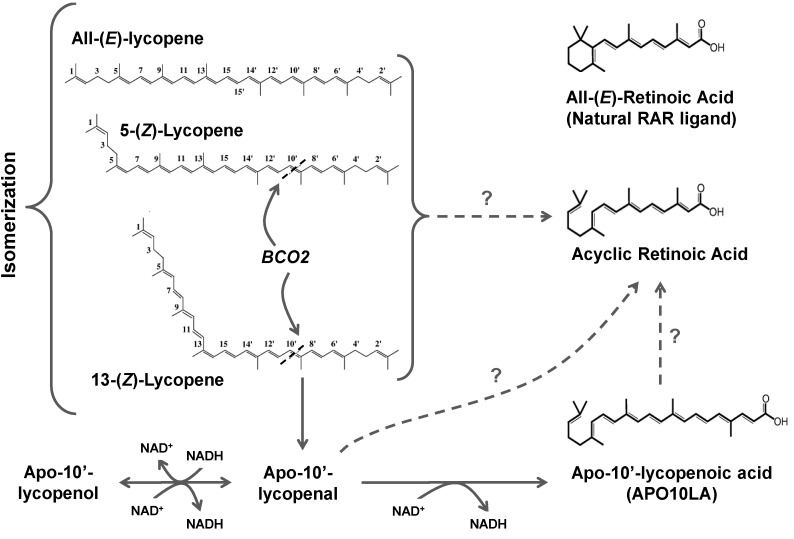
Schematic illustration of lycopene metabolic pathway by β carotene-9′,10′-oxygenase (BCO2). 5-(*Z*) and 13-(*Z*) lycopene are preferentially cleaved by BCO2 at 9′10′-double bond. The cleavage product, apo-10′-lycopenal, can be further oxidized to apo-10′-lycopenol or reduced to apo-10′-lycopenoic acid (APO10LA), dependent on the presence of NADH. Whether apolycopenoids can be oxidized to form acyclic retinoids remains to be elucidated. RAR, retinoic acid receptor.

Complex metabolism of lycopene provides the rationale that lycopene metabolites may also exhibit biological effects [136,140]. Due to lycoepene’s association with a decreased risk for chronic diseases in humans, considerable attention is paid on the study of lycopene’s metabolism, and has since made significant strides. Lycopene metabolism involves both the chemical and enzymatic modifications to generate a collection of lycopenoids that include lycopenal, lycopenol and lycopenoic acid (Figure 3). These lycopenoids are generated in plants and animals through oxidation [136,155], non-enzymatic and enzymatic cleavage of the double bonds within the lycopene molecule [136,140,155]. Thus the lycopenoids detected in human plasma maybe from dietary sources as well as from endogenous synthesis [138].

#### 3.3.1. Chemical Oxidation

The unique structures of polyisoprenoid and carbonyl compounds such as lycopene make them susceptible to cleavage by auto-oxidation, radical-mediated oxidation, and singlet oxygen [156]. These oxidative conditions can be present both synthetic conditions (food processing), as well as *in vivo* if the tissues are exposed to oxidative stress, including those observed in the obese state. Utilizing various oxidizing systems and *in vitro* experiments, scientists have demonstrated lycopene’s susceptibility to cleavage, and identified unique oxidative products of lycopene. After incubating lycopene under atmospheric oxygen at 37 °C for 72 h, Kim *et al.* identified eight oxidative products (3,7,11-trimethyl-2,4,6,10-dodecatetraen-1-al,6,10,14-trimethyl-3,5,7,9,13-pentadecapentaen-2-one, acyclo-retinal, apo-14′-lycopenal, apo-12′-lycopenal, apo-10′-lycopenal, apo-8′-lycopenal, and apo-6′-lycopenal) [157]. Utilizing another oxidizing system, Caris-Veyrat *et al.* identified eight apolycopenals and three apolycopenones generated from oxidation of lycopene [158]. Among these metabolites included acyclo-retinal and apo-10′-lycopenal, which are the predicted central and eccentric enzymatic cleavage products of lycopene respectively [158]. The synthesis of acyclo-retinoids (ACR) has generated significant research interests. Acyclo-retinoic acid has been demonstrated to have chemopreventative effects against liver cancer (reviewed below), and acyclo-retinal could be further oxidized to acyclo-retinoic acid when incubated with pig liver homogenate [157], providing evidence for the potential *in vivo* synthesis. Commercial processing of tomatoes can generates lycopenoids through auto-oxidation [159], including six apolycopenals, eight lycopene epoxides and a cyclolycopene diol.

#### 3.3.2. Oxidative Metabolism in Plants

In plants, lycopene is synthesized as precursors of other carotenoids including carotenes [160], but may also be further metabolized to form lycopenoids (apolycopenoids and epioxides derivatives) as found in both raw [138,161,162,163,164], and processed tomato products [138,159,160,165]. Kopec *et al.* recently quantified apolycopenals (apo-6′-, 8′-, 10′-, 12′-, and 14′-lycopenal) in fruits and vegetables, and found the sum of the lycopenals in Roma tomato and tomato paste were 65 ng/g and 734 ng/g respectively [138]. The presences of lycopenoids in plants have been attributed partly to two different classes of carotenoid cleavage enzymes. The 9-*cis*-epoxycarotenoid dioxygenases are responsible for the formation of the plant hormone abscisic acid from neoxanthin and violaxanthin, and the second class of enzymes is called carotenoid cleavage dioxygenase (CCD). Schwartz *et al.* have identified nine potential carotenoid cleavage enzymes from the analysis of the Arabidopsis genome [166,167,168]. CCD1 and CCD7 have been demonstrated to cleave the 9′,10′ double bond of lycopene, and CCD1 can also cleave the 5′,6′ double bond [169]. It remains to be determined as to whether other CCDs can effective cleave lycopene in plant tissue.

#### 3.3.3. Oxidative Metabolism in Mammals

Mammalian lycopene metabolism has been attributed to the activities of endogenous oxidants, as well as to the reactions with the mammalian version of carotenoid cleavage enzymes. A series of *in vitro* experiments provided evidence to indicate potential *in vivo* formation of lycopene metabolites. For example, incubating deuterated lycopene with rat intestinal post-mitochondrial fractions generated 3-keto-apo-13-lycopenone and 3,4-dehydro-5,6-dihydro-15,15′-apolycopenal, as well as four oxidative metabolites [155]. Only two carotenoid cleavage enzymes have been discovered in mammals, and controversy exists in regards to their capacities in metabolizing lycopene [140].

##### 3.3.3.1. BCO1

The β carotene 15′,15′-oxygenase (BCO1) has been cloned and characterized in a number of mammalian species, and it is responsible for the central cleavage of carotenoids at the 15′,15′ double bond [170,171,172,173]. For pro-vitamin carotenoids such as α-carotene, β-carotene, and β-cryptoxanthin, central cleavage by BCO1 is the major pathway for vitamin A synthesis [173]. However, it remains controversial as to whether lycopene is a potential substrate for BCO1. Redmond *et al.* observed the synthesis of central cleavage product acyclo-retinal when lycopene was incubated in recombinant murine BCO1, but acyclo-retinal was only detected when lycopene concentrations were 2.5–3 times higher than the observed Km for β-carotene [174]. Most recently, Sena *et al.* observed that lycopene was able to be cleaved by the purified recombinant human BCO1 to yield acyclo-retinal, but this enzymatic reaction has a relatively lower *V*_max_ (28.0 ± 0.8 nmol acyclo-retinal/mg BCO1/h) and *K*_M_ (1.7 ± 0.4 μM), in comparison to the cleavage of β carotene by BCO1 (*V*_max_ = 197.2 ± 23.2 retinal/mg BCO1/h; *K*_M_ = 17.2 ± 4.0 μM) [175]. In contrast, numerous studies found no detectable activity of BCO1 towards lycopene. Utilizing a purified recombinant BCO1 isolated from human liver cDNA library, Lindqvist and Andersson demonstrated that BCO1 can cleave both β-carotene and β-cryptoxanthin, but not lycopene [173]. The low affinity of BCO1 towards β-cryptoxanthin as compared to β-carotene lead the authors to conclude that the presence of at least one unsubstituted β-ionone ring is required for the effective catalytic cleavage by BCO1 [173]. Other investigators have also observed no lycopene cleavage by human BCO1 from the retinal pigment epithelium [176], by the Drosohpila homologue of BCO1 [171], and the crude preparations of rat liver and intestine [177]. These studies have provided a strong rationale that lycopene is a poor substrate for BCO1, but it is important to note that these mechanistic studies most likely utilized All-(*E*)*-*lycopene. All-(*E*) isomer predominates the main dietary sources of lycopene, but a significant greater portion of lycopene found in mammalian tissues are in the (*Z*)-conformation [140,178]. Using dietary ^14^C-lycopene, Ross *et al.* demonstrated that 92% all-trans lycopene is extensively metabolized in humans to 50% All-(*E*)*-*, 38% 5-(*Z*)-, 1% 9-(*Z*)- and 11% other (*Z*)-lycopene isomers after 24 h [178]. Future investigations are required to elucidate whether isomerization of lycopene may impede BCO1 capacity to metabolize this carotenoid.

##### 3.3.3.2. BCO2

β Carotene-9′,10′-oxygenase (BCO2) is the second mammalian carotenoid cleavage enzyme first cloned and characterized by Kiefer *et al.*, and is responsible for the asymmetric (eccentric) cleavage of carotenoids at the 9′,10′ double bond [179]. Using isolated murine BCO2, Kiefer *et al.* demonstrated the BCO2-dependent cleavage of β-carotene and lycopene [179]. Our laboratory has later cloned and characterized the BCO2 enzyme from ferrets (*Mustela putorius furo*), a mammal that share similar carotenoid metabolism as humans (absorption, tissue distribution and concentration) [180]. Further analysis of the recombinant ferret BCO2 revealed that BCO2 has a higher optimum pH than BCO1 [180]. This observation supports the later findings by Amengual *et al.* that BCO2 is a mitochondrial protein, whereas BCO1 is localized in the cytoplasm [181]. BCO2 appears to have much broader substrate specificity than BCO1 in regards to the β-ionone ring structure on carotenoids. Utilizing *E. coli* expressing the recombinant murine BCO2, Amengual *et al.* observed BCO2 catalysis of various carotenoids at the 9′,10′ position, including β-carotene, β-cryptoxanthin, lutein and zeaxanthin [181]. Apocarotenoids generated from BCO2-mediated cleavage of lutein and zeaxanthin were also able to be further metabolized by BCO2 into rosafluene [181]. Our laboratory observed that recombinant ferret BCO2 can catalyze the cleavage of All-(*E*)-β-carotene and (*Z*)-lycopene isomers (5-(*Z*)-lycopene and 13-(*Z*)-lycopene) effectively at the 9′,10′ double bond, generating β-apo-10′-carotenal and β-apo-10′-lycopenal respectively (Figure 3) [180]. However, all-(*E*)-lycopene was not an effective substrate for the recombinant ferret BCO2, providing some evidence to suggest that lycopene isomerization may alter its affinity for mammalian carotenoid cleavage enzymes. Lycopene was also observed to accumulate more significantly in mice that lack the BCO2 enzyme than wild-type mice [182], further support that BCO2 is an important enzyme involved in lycopene metabolism. BCO2 is differentially expressed in numerous human tissues [183], thus the localized apolycopenoids concentration may vary at the tissue level. 19 Single-nucleotide polymorphism (SNP) of BCO2 have been found in humans that may alter its expression and/or function of this enzyme depending on the modification of reading frame [184]. The SNP rs2115763 at the BCO2 locus was associated with IL-18 concentration [184,185], a pro-inflammatory cytokine that correlated with diabetes and cardiovascular disease. Female variant allele carriers of a common SNP in the BCO2 gene can also have reduced fasting HDL-cholesterol concentrations [184,186]. In other mammals, BCO2 SNPs have been described in bovine [187,188], sheep [189], and chickens [190]. The nonsense mutation of BCO2 in bovine has been shown to affect β-carotene concentration in plasma, milk and adipose tissue [187,188]. Other polymorphisms of the BCO2 gene are related to adipose color in lamb [189], or skin color in chickens [190]. Therefore, it is conceivable to hypothesize that a gene-diet interaction with respect to human health and disease may exist between the BCO2 enzyme and dietary carotenoids.

##### 3.3.3.3. Potential Alternative Pathways

It has been well-documented that anoxygenic photosynthetic bacteria synthesize carotenoids, produce carotenoid-derived volatile compounds, and express carotene oxygenases that resemble those involved in plant abscisic acid synthesis [191]. Questions remain as to whether the human gut microbiome can synthesize apolycopenoids from dietary lycopene, and/or modulate lycopene metabolism at the intestinal mucosa. Since gut microbiome can be modulated through diet, and is significantly affected by disease states including metabolic syndrome and NAFLD [22,23,24,25,26,27], it would be of importance to investigate the crosstalk between these parameters and human carotenoid metabolism.

#### 3.3.4. Oxidation of Apolycopenoids

The apolycopenoids generated from the BCO2 cleavage still share the similar structure and oxidation susceptibility as their parent compound lycopene, and can potentially evolve and form other metabolites. We observed that apo-10′-lycopenal can be further metabolized to form apo-10′-lycopenol and apo-10′-lycopenoic acid (APO10LA) when incubated in post-nuclear fraction of ferret liver homogenates, depending on the presence of NADH or NAD^+^ respectively (Figure 3) [180]. Ross *et al.* observed that dietary ^14^C-lycopene was rapidly metabolized in humans into polar metabolites and excreted into urine, with the rapid peak of ^14^CO_2_ in breath after dosing, suggesting that β-oxidation is involved in lycopene metabolism [178]. Rats fed ^14^C-lycopene resulted in apo-8′- and apo-12′-lycopenal in hepatic tissue 24-h post-dosing, plus a significant amount of unidentified short-chain polar metabolites present differentially in tissues (20%, 38% and 67% of isotope label in liver, seminal vesicle and prostate respectively) [192]. No APO10LA was detected in white adipose tissue of mice orally treated with lycopene or APO10LA using mass spectrometry, but observed a metabolite with a molecular weight of 2 Da larger than APO10LA, suggesting further metabolism of APO10LA might have occurred [193]. Since BCO1 has been shown to cleave and metabolize β-apocarotenoids [194], further investigation is warranted to determine whether these polar metabolites from lycopene supplementation are BCO1/BCO2 cleavage products of apolycopenoids, or generated by *in vivo* carotenoid cleavage enzymes that have yet to be identified. Apo-carotenoids generated from eccentric cleavage of β-carotene have been shown to undergo β-oxidation to produce retinoic acid (RA), a molecule that can modulate cell differentiation though transactivation of RA receptor elements (RAREs) [195]. It remains to be elucidated as to whether apolycopenoids can be oxidized to form acyclic retinoids (ACR), the hypothesized central cleavage product of lycopene (Figure 3). ACR has been shown to induce RA receptor beta (RARβ), a molecule where its transcription is modulated by RARE [75,196]. APO10LA has been shown to share similar RARβ induction as ACR [193,197]. However, further research is required to determine whether this observed RARβ-induction is a direct effect of APO10LA.

### 3.4. Effects of Apolycopenoids

Recent investigations including our own have proposed that the biological activities of lycopene may be mediated in part by lycopene’s metabolites [193,197,198,199]. As previously mentioned, genetic variants of the BCO2 gene are prevalent in humans and other mammals [184], and BCO2 polymorphism has been associated with modified status of carotenoid and pro-inflammatory cytokine IL-18 [182,184,187,200]. Murine models deficient in BCO2 enzyme altered mitochondrial function and induced lipid accumulation in liver tissue, as well as elevated oxidative stress in liver cells when treated with lutein or zeaxanthin [181]. Further, zeaxanthin supplementation in BCO2 deficient mice resulted in the induction of stress pathways in hepatic tissue, including MAPK signaling [181]. This observation demonstrated the potential gene and nutrient interactions between BCO2 and lycopene on metabolic consequences, and could potentially explain the inconsistent clinical results in lycopene’s beneficial effects.

Despite the lack of evidence for the *in vivo* synthesis of ACR from lycopene, ACR has received considerable attention in the study of HCC chemoprevention. ACR has been demonstrated to inhibit both carcinogen-induced and spontaneously occurring hepatocarcinogenesis *in vivo* [75,201,202,203]. ACR treatments on hepatoma cell lines can suppress growth through inducing of apoptosis via caspase-3 cleavage [204], inhibiting RXRα phosphorylation by suppressing Ras-MAPK pathway [196], up-regulating RARβ expression [75,205], and promoting cell cycle arrest by increasing cellular levels of p21^CIP1^ [205,206,207]. ACR treatments can inhibit angiogenesis of endothelial cells by modulating the mitogenic MAPK pathway [208]. Oral ACR in patients with HCC significantly reduced cancer recurrence, and improved survival without serious side-effects [209,210]. A phase II/III clinical trial of ACR was also effective in preventing second primary HCC in hepatitis C virus-positive patients [211]. Whether ACR treatment is effective in preventing NAFLD/NASH associated HCC and reversing NASH pathologies in humans remains to be investigated. However, a recent study utilizing genetically-induced obese male mice (*db*/*db*) showed that 34 weeks of ACR supplementation at pharmacological doses (0.03% and 0.06% diet) significantly inhibited DEN-induced liver cell adenomas development [75]. This observed chemopreventative effects of ACR was associated with attenuated hepatic steatosis, insulin resistance as measured by QUICKI value, and markers of chronic inflammation [75]. Future studies need to be conducted to address whether ACR supplementation at a physiological dose would suppress diet-induced hepatic injuries associated with NAFLD/NASH.

### 3.5. Potential Molecular Mechanisms

It remains to be determined as to whether lycopene is an important nutrient with health benefits, specifically its efficacy in inhibiting obesity-promoted liver tumorigenesis *in vivo* and in humans. However, increasing *in vivo* and *in vitro* evidence support that lycopene has multi-faceted biological functions. The following provides an update on the potential mechanisms by which lycopene and its metabolites may exhibit chemopreventative effects against liver cancer.

#### 3.5.1. Modulating Pro-Inflammatory Signaling and Cytokine Expression

Pro-inflammatory NF-κB/STAT3/AP-1 signaling activation is involved in the production of tumor-promoting cytokines by immune/inflammatory and other associated cells, thereby stimulating cell proliferation and survival of transformed cells [7]. The mitogenic MAPK-signaling cascade can also modulate pro-inflammatory and survival when activated by physical stress and receptor mediated mechanisms [66,67]. Lycopene has been shown to inhibit NF-κB and MAPK signaling in numerous studies through multiple mechanisms [212,213,214,215,216]. In LPS-exposed macrophages, lycopene treatment (1 µM) inhibited NF-κB and MAPK-Erk activation, as well as decreased TNFα production, potentially though inhibiting LPS-induced superoxide synthesis [212]. Marcotorchino *et al.* also observed that lycopene pretreatment (0.5–2 µM) on RAW 264.7 macrophages appeared to have dose-dependently reduced LPS-stimulated TNFα mRNA and protein expression [217]. These anti-inflammatory effects of lycopene was associated with decreased JNK and NF-κB signaling activation, as well as with reduced LPS-stimulated macrophage migration [217]. Similar NF-κB-inhibiting effects of lycopene (0.5–2 µM) were observed in cigarette smoke-stimulated macrophages, and the inhibitory mechanisms involved the suppression of NF-κB DNA binding, reduction in NF-κB/p65 nuclear translocation, as well as the inhibition of NF-κB inhibitors IKKα and IκBα by phosphorylation [213].

Lycopene capacity to inhibit NF-κB signaling also occurs in cells other than of myeloid origin. Of relevance to NAFLD and obesity, it was observed that pretreatment of preadipocytes, differentiated 3T3-L1 adipocytes, and human adipocyte primary culture with lycopene (2 µM) decreased macrophage conditioned medium induced and TNFα-mediated induction of pro-inflammatory cytokine and chemokines (IL-6 and MCP-1) [214,217]. TNFα is a known cytokine to induce NF-κB signaling [7,46]. Using a transient transfection system where 3T3-L1 adipocytes were transfected with the NF-κB-dependent luciferase reporter plasmid, Gouranton *et al.* showed that lycopene pretreatment significantly reduced the TNFα-stimulated reporter luciferase activity [214], suggesting that lycopene inhibited cytokines production through suppressing NF-κB activation. Further, lycopene pretreatment in adipose tissue explants of mice subjected to HFD for 6 weeks decreased proinflammatory cytokine and chemokine expression [214]. In a separate study, dietary lycopene in diet-induced obese rats significantly reduced adipose tissue expression of IL-6 and MCP-1 mRNA expression [218], supporting the notion that lycopene may suppress inflammation associated with metabolic excess. More specific to transformed liver cells, lycopene treatments (1–10 µM) on SK-Hep-1 cells from human hepatoma significant reduced NF-κB activation and promoter-binding, partially through up-regulation of IκBα protein expression [219].

Limited research has been conducted to elucidate the biological activities of lycopene metabolites, but evidence from recent studies suggests that apolycopenoids may suppress expression of pro-inflammatory cytokines. It has been shown that APO10LA treatment (2 µM) decreased pro-inflammatory *Il-6* and *Il-1β* mRNA expression in TNFα-stimulated adipocytes (3T3-L1 cells; adipose tissue explants of mice subjected to HFD in *ex vivo* culture; primo culture of human mature adipocytes *in vitro*), but did not alter markers of adipogenesis [193]. APO10LA and apo-14′-lycopenoic acid (2 µM) pretreatments on human THP-1 macrophages suppressed the H_2_O_2_-induced NF-κB and MAPK activation [220], and apo-14′-lycopenoic acid appeared to have greater NF-κB/MAPK-suppressing capacity than APO10LA. It is important to note that the effective APO10LA concentrations to exhibit the described effects *in vitro* were higher than the reported human plasma lycopene concentration (50–900 nmol/L) [221]. However, studies by our laboratory found that the intracellular APO10LA concentration was below detection level [197,222]. The absence of intracellular APO10LA could be due to the low carotenoids uptake in cell culture study. We also previously showed that dietary lycopene is better accumulated in tissue than in plasma [222,223]. This may partially explain why the effective APO10LA concentrations for *in vitro* studies were much higher than plasma concentration.

**Figure 4:**
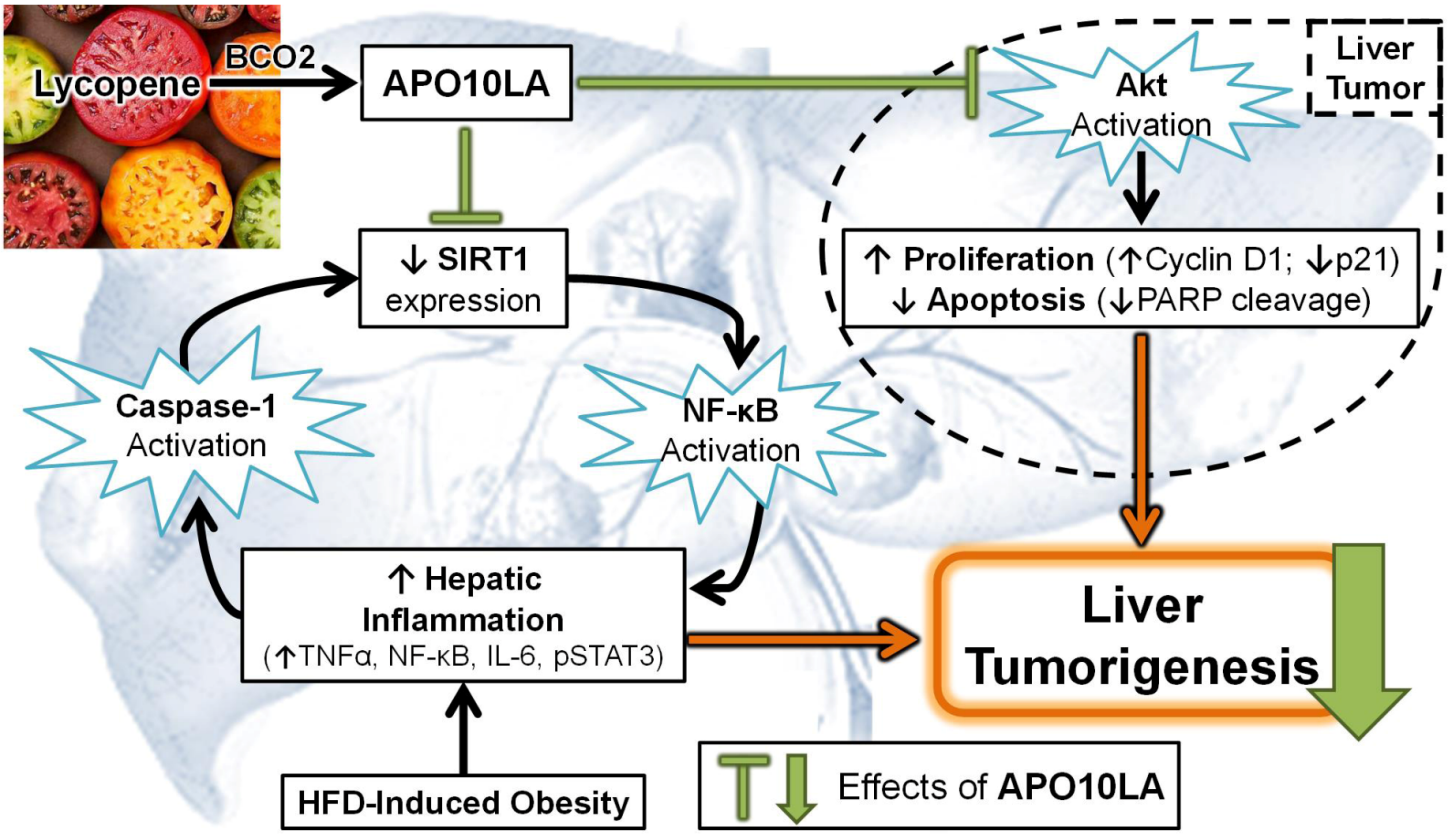
Schematic diagram of potential mechanisms by which apo-10′-lycopenol acid (APO10LA) inhibits high-fat diet (HFD)-promoted hepatic inflammation and tumorigenesis. Lycopene is endogenously metabolized to APO10LA by BCO2. APO10LA inhibits HFD-induced hepatic inflammation and tumorigenesis, through interrupting the vicious cycle of HFD-promoted up-regulation of nuclear factor-κB (NF-κB) signaling, escalation in inflammasome function and reduction in sirtuin 1 (SIRT1) signaling. APO10LA reduced HFD-promoted hepatic tumor number and volume by inhibiting Akt activation in liver tumors. IL, interleukin; PARP, poly ADP ribose polymerase; STAT3, signal transducer and activator of transcription 3; TNFα, tumor necrosis factor alpha.

A recent study conducted in our laboratory showed that dietary APO10LA at 10 mg/kg diet significantly reduced hepatic inflammation (decreased inflammatory foci, TNFα, IL-6, NF-κB p65 protein expression and STAT3 activation) and tumorigenesis in HFD-fed obese mice (schematics of results in Figure 4) [224]. Moreover, reduction in NF-κB p65 protein in response to APO10LA supplementation was significantly correlated with decreased liver tumor volume and IL-6 protein expression [224], suggesting lycopene metabolites may exhibit protective effects against obesity associated hepatic inflammation and associated tumorigenesis.

#### 3.5.2. Antioxidant Mechanism

As a polyisoprenoid, lycopene’s unique structure consists a series of centrally located conjugated double bonds [156]. These double bonds can contribute to the chemical reactivity towards free radicals and oxidizing agents that may be relevant for lycopene antioxidant effects in animals [225,226,227]. DNA damage by oxidative stress including ROS is one of the key steps for cancer initiation. Apart from chemically interacting with radicals, lycopene can also reduce oxidative stress by modulating ROS-producing enzymes (CYP-P450 enzymes, NADPH oxidase, iNOS, COX-2 and 5-LOX), and inducing antioxidant/detoxifying Phase II enzymes (HO-1, NQO1 and GST). These Phase II enzymes are regulated by the nuclear factor E_2_-related factor 2-antioxidant response element (Nrf2-ARE) system. Lycopene antioxidant effects and associated mechanisms have been extensively reviewed [136,140,228,229]. Toledo *et al.* observed that the chemopreventative capacities of lycopene against hepatic precancerous markers were associated with decreased hepatic DNA strand breakage [153], and Astorg *et al.* showed that dietary lycopene significantly decreased hepatic preneoplastic lesions by potentially inhibiting CYP2E1 [151]. Investigation by our laboratory revealed that dietary lycopene and tomato extract were also effective in inhibiting NASH-promoted, DEN-initiated hepatocarcinogenesis in rats, but through different mechanisms observed by Astorg *et al.* [139,151]. Our study conducted by Wang *et al.* observed that tomato extract inhibited NASH-promoted hepatocarcinogenesis through reduction in CYP2E1 expression, whereas the chemopreventative effects of lycopene were associated with the induction of Nrf2 and HO-1 [139]. These results suggest the potential differential chemopreventative effects between the whole food tomatoes that contain apolycopenoids, *versus* the isolated carotenoid lycopene.

The antioxidant capacity of apolycopenoids has been observed in BEAS-2B human bronchial epithelial cells, where pretreatment of BEAS-2B cells with APO10LA resulted in a dose-dependent inhibition of both ROS production and H_2_O_2_-induced oxidative damage [222]. With respect to mechanisms involved in NAFLD progression, human HepG2 cells treated with apo-8′-lycopenal (1–10 µM) dose-dependently induced Nrf2-ARE activity, and the expression of HO-1 and NQO1 [230]. These Nrf2-inducing effects were found to be depending on suppressing Erk/p38 activation by phosphorylation, and Nrf2 inhibitor Kelch-like ECH-associated protein 1 (Keap1) [230]. APO10LA and apo-14′-lycopenoic acid (2 µM) pretreatments on human THP-1 macrophages also reduced H_2_O_2_-induced ROS production [220]. These observations were accompanied by the reduction in Erk/p38 activation, and the ROS producing enzyme COX-2 [220]. A recent study from our laboratory showed that APO10LA supplementation (10 mg/kg diet) regulated HO-1 protein expression in HFD-fed BCO2 knockout mice [231]. However, subsequent investigations are required to determine whether the antioxidant capacity of apolycopenoids is a significant mechanism in suppressing NAFLD progression.

#### 3.5.3. Retinoid Receptors Interactions

The central cleavage BCO1 enzyme is responsible for generating vitamin A from provitamin A carotenoids. Vitamin A derivatives include all*-*(*E*)-RA and 9′-(*Z*)-RA are ligands for retinoid receptors RAR and RXR respectively [76]. Both RAR and RXR consist of three types (α, β, and γ), with different isoforms for each of the types [76]. Retinoid receptors are ligand-dependent transcription factors that regulate genes involved in critical processes in human physiology including differentiation and metabolism, whereas the loss in retinoid activity results in deviations from normal cell proliferation and death [76]. These gene transcription regulations require retinoid receptors to form dimerized complexes and subsequently bind to RARE/RXRE that are located in the 5′ promoter region of responsive genes [76]. With respect to cancer cells, up-regulating retinoid receptors expression and activity can mediate growth inhibitory effects. Some of these RAR/RXR-induced growth inhibitory genes include the cell cycle inhibitor p21^CIP1^ and RARβ [76]. RARβ appears to have the dominant tumor suppressor role, whereas loss of RARβ expression is associated with tumor progression. RARβ expression is often decreased in human HCC and liver cancer cell lines [196], and suppressed in HCC lesions in rats with chemically-induced HCC [232]. Phosphorylation of RXRα inhibits retinoid signaling, and has been shown to accumulate in human HCC tissue and cell lines [196]. This RXRα phosphorylation site is found to be a MAPK/Erk consensus site [77], and as previously mentioned, elevated MAPK/Erk signaling is often associated with metabolic syndrome and insulin resistance, thus this provides a potential mechanistic link between metabolic syndrome and increased HCC risk. Further, inducing hepatic retinoid signaling in mice by eliminating lecithin:retinol acyltransferase expression resulted in protection against chemically-induced HCC. This evidence suggests that retinoid signaling plays a protective role against HCC development.

Utilizing a series of luciferase reporter constructs with and without site-directed mutagenesis of the RAR binding site of RARE, our laboratory has demonstrated that APO10LA increased luciferase activity only in wild-type RARE, although its activity was at a magnitude less than all*-*(*E*)*-*RA [197]. This study raised an important question as to whether APO10LA can also function as a retinoic acid analog. Indeed, using the RARE-luciferase mouse model, a study showed that APO10LA can transactivate RAR *in vivo* and *in vitro*, as well as induce RAR target genes (RARβ and CYP26A1) in adipose tissue [193]. Further transcriptomics analysis revealed that APO10LA treatment modulated 27.5% of the genes in adipose tissue that were also regulated by all*-*(*E*)*-*RA, the known ligand of RAR [193]. These data suggest that lycopene metabolites may elicit differential biological activities than their parent compound. The metabolism of lycopene and the subsequent generation of apolycopenoids might be required to exhibit lycopene’s effects on RAR/RXR-dependent gene transcription that are similar to other natural ligands of RAR and RXR.

#### 3.5.4. Anti-Metastatic Effects

Tumor metastasis is the major cause of cancer recurrence and mortality, involving complex and multifaceted program that allows malignant cells to disseminate from primary tumors and to invade a distance site. Increased intra-tumoral blood supply is essential for sufficient growth of large tumors, provides a route for distance metastasis, and it is a process mediated by angiogenesis. Angiogenic mediators including vascular endothelial growth factor (VEGF) are involved in orchestrating the development of new vessels, and are up-regulated by hypoxia as well as by oncogenic signals [233]. Metastatic cells acquire invasion capacity by expressing elevated levels of MMPs that can degrade all know components of ECM [233]. MMP2 and MMP9 are closely related to metastasis, and MMP9 expression has been correlated with the growth of human HCC [234]. MMP9 has also been shown to release and activate latent forms VEGF ligands sequestered within the ECM, thereby facilitating angiogenesis [233].

Lycopene treatments (1–10 µM) on SK-Hep-1 cells significant suppressed *in vitro* cell invasion [219]. This observed property of lycopene was associated with the reduction in MMP9 gene and protein expression, as well as the elevation in tissue inhibitor of metalloproteinase-1 (TIMP-1) expression, which inactivates MMP9 through post-translational regulation. A follow-up study from the same research group showed that oral lycopene supplementation (20 mg/kg BW) significantly reduced the expression of VEGF, MMP9, and suppressed lung metastasis in athymic nude mice injected with SK-Hep-1 cells [149], providing further evidence to suggest that lycopene may have anti-metastatic effects on liver tumors.

Lycopene metabolite apo-8′-lycopenal has been shown to have similar anti-metastatic effects in SK-Hep-1 cells as lycopene treatments [198], and sharing comparable mechanisms including MMP9 suppression and elevation in TIMP1. However, apo-8′-lycopenal treatment was more effective than lycopene at the same concentration (10 µM) [198]. Recently, we observed that APO10LA is effective at inhibiting migration and invasion of both cancer and endothelial cells by suppressing actin remodeling and ruffling/lamellipodia formation [235]. This APO10LA-mediated inhibition of endothelial cell migration/invasion suppressed MMP2 expression and angiogenesis, as measured by endothelial cell tube formation and aortic ring assays. These accumulated results indicate apolycopenoids may exhibit anti-metastatic properties.

#### 3.5.5. SIRT1 Up-Regulation

Persistent activation of hepatic JNK signaling can inhibit SIRT1 expression and activity [236,237]. As mentioned previously, SIRT1 activation is recognized to protect against obesity-induced hepatic steatosis and inflammation [48,53,54,55,56]. The genetic overexpression of SIRT1 has also been shown to protect mice from obesity-promoted hepatocarcinogenesis [50], suggesting that SIRT1 is a potential molecular target for dietary and therapeutic agents against HCC development. SIRT1 activation is associated with AMP-activated protein kinase activity, a central metabolic sensor with its activity controlled by the tumor suppressor liver-kinase B1 [48,49,50,51,52]. SIRT1 has also recently been shown to negatively regulate growth hormone-dependent IGFs expression in the liver through STAT5 deacetylation [238], which can potentially modulate IGF-dependent oncogenic signaling.

SIRT1 can deacetylate a number of non-histone targets to regulate metabolism and inflammation [48,49,50,51,52]. SIRT1 activation is recognized to protect against obesity-induced glucose intolerance, insulin resistance, hepatic steatosis, inflammation, and carcinogenesis [48,50,53,54,55,56]. SIRT1 agonist resveratrol treatment has also been shown to improve health and survival of mice on a high calorie diet [239]. SIRT1 modulates steatosis development through inhibiting the expression of lipogenic transcription factor SREBP-1c, and increasing mitochondrial biogenesis by modulating PGC-1α acetylation [240]. SIRT1 attenuates hepatic inflammation by deacetylating and/or reducing NF-κB p65 expression, thereby attenuating NF-κB-induced expression of pro-inflammatory cytokines [48,50,52,53,241]. A recent study demonstrated SIRT1 capacity to attenuate hepatic IGF expression through STAT5 deacetylation [238], further supports the notion that SIRT1 can be an effective therapeutic target in suppressing NAFLD progression.

To our knowledge, there are no published studies that investigated or observed lycopene modulations on SIRT1. Preliminary data generated from our laboratory revealed a novel role of APO10LA in up-regulating hepatic expression of SIRT1, decreasing acetylation of SIRT1 downstream target, and inhibiting steatosis in genetically-induced obese (*ob*/*ob*) mice [199]. Very recently, we observed similar effects in our diet-induced obesity model, where APO10LA supplementation (10 mg/kg diet) for 24 weeks significantly induced hepatic SIRT1 protein expression and deacetylation of NF-κB p65 in HFD-fed mice (Figure 4) [224]. Our data suggested that APO10LA up-regulated SIRT1 protein expression by inhibiting HFD-induced activation of hepatic caspase 1 [224]. APO10LA-mediated SIRT1 induction was associated with reduced hepatic IL-6 and TNFα protein expression, and more importantly, the APO10LA’s anti-inflammatory effects were inversely correlated with hepatic tumorigenic outcomes [224]. Together, this evidence supports the notion that APO10LA can inhibit HFD-promoted hepatic inflammation and tumorigenesis in mice by interrupting the vicious cycle of HFD-promoted up-regulation of NF-κB signaling, escalation in inflammasome function and reduction in SIRT1 signaling [224]. Further investigations with SIRT1 knockout mice are currently ongoing in our laboratory to elucidate whether APO10LA’s chemopreventative effects are SIRT1-dependent.

## 4. Conclusions

The scientific research community on carotenoids has made significant strides on the understanding of lycopene metabolism, and the biological effects of lycopene and metabolites apolycopenoids. There is strong evidence to suggest that BCO2-mediated vertebrate carotenoid metabolism may play a critical role in lycopene’s physiological effects, since modifying BCO2 enzyme expression in animal can alter biological implications of non-provitamin A carotenoids. This notion is supported by the demonstration that apolycopenoids are biologically active. In particular, we and others have provided the evidence that APO10LA was effective in up-regulating SIRT1 protein expression, activating the Nrf2-mediated expression of HO-1, transactivating the RARE-mediated RARβ induction, as well as in protecting mice against carcinogenesis in lung and liver. Intriguingly, the chemical structure of APO10LA is also similar to acyclic retinoic acid (Figure 3), of which has recently been shown to prevent liver fibrosis and HCC development. Moreover, genetic variants of the BCO2 gene are prevalent in humans and other mammals. BCO2 polymorphism has been associated with the alterations in human and animal carotenoid status, pro-inflammatory cytokine IL-18 expression, and in fasting HDL levels. These observations suggest that the existence in BCO2 polymorphism and its potential impact on lycopene metabolism may partially explain the conflicting outcomes of clinical trials with lycopene.

In addition, recent studies provided direct evidence that SIRT1 can protect against carcinogenesis associated with metabolic syndrome through its anti-inflammatory effects. Thus, the development of dietary SIRT1 agonists will provide useful interventions to suppress inflammation and carcinogenesis associated with metabolic alterations. We have recently provided experimental evidence to suggest that SIRT1 is a potential molecular target of carotenoids action. These notions clearly warrant further evaluation.
